# Endothelial cell-derived nidogen-1 inhibits migration of SK-BR-3 breast cancer cells

**DOI:** 10.1186/s12885-019-5521-8

**Published:** 2019-04-04

**Authors:** Daniela A. Ferraro, Francesca Patella, Sara Zanivan, Cinzia Donato, Nicola Aceto, Monica Giannotta, Elisabetta Dejana, Maren Diepenbruck, Gerhard Christofori, Martin Buess

**Affiliations:** 1 Tumor Biology, Department of Biomedicine, University of Basel, 4058 Basel, Switzerland; 2 Tumour Microenvironment and Proteomics, Cancer Research UK Beatson Institute, Glasgow, G611BD UK; 3 Cancer Metastasis, Department of Biomedicine, University of Basel, 4058 Basel, Switzerland; 4 Vascular Biology Unit, FIRC Institute of Molecular Oncology, 20139 Milan, Italy; 5 Department of Oncology, St. Claraspital, Kleinriehenstrasse 30, 4016 Basel, Switzerland

**Keywords:** Tumor stroma, Endothelial cells, Breast cancer

## Abstract

**Background:**

The tumour microenvironment is a critical regulator of malignant cancer progression. While endothelial cells have been widely studied in the context of tumour angiogenesis, their role as modulators of cancer cell invasion and migration is poorly understood.

**Methods:**

We have investigated the influence of endothelial cells on the invasive and migratory behaviour of human cancer cells in vitro.

**Results:**

Upon exposure to culture supernatants of endothelial cells, distinct cancer cells, such as SK-BR-3 cells, showed significantly increased invasion and cell migration concomitant with changes in cell morphology and gene expression reminiscent of an epithelial-mesenchymal transition (EMT). Interestingly, the pro-migratory effect on SK-BR-3 cells was significantly enhanced by supernatants obtained from subconfluent, proliferative endothelial cells rather than from confluent, quiescent endothelial cells. Systematically comparing the supernatants of subconfluent and confluent endothelial cells by quantitative MS proteomics revealed eight candidate proteins that were secreted at significantly higher levels by confluent endothelial cells representing potential inhibitors of cancer cell migration. Among these proteins, nidogen-1 was exclusively expressed in confluent endothelial cells and was found to be necessary and sufficient for the inhibition of SK-BR-3 cell migration. Indeed, SK-BR-3 cells exposed to nidogen-1-depleted endothelial supernatants showed increased promigratory STAT3 phosphorylation along with increased cell migration. This reflects the situation of enhanced SK-BR-3 migration upon stimulation with conditioned medium from subconfluent endothelial cells with inherent absence of nidogen-1 expression.

**Conclusion:**

The identification of nidogen-1 as an endothelial-derived inhibitor of migration of distinct cancer cell types reveals a novel mechanism of endothelial control over cancer progression.

**Electronic supplementary material:**

The online version of this article (10.1186/s12885-019-5521-8) contains supplementary material, which is available to authorized users.

## Background

The tissue microenvironment is critical for normal development and homeostasis. Considering normal breast tissue, for example, mouse mammary epithelial cells isolated from mid-pregnancy mammary glands form acinus-like structures if grown on a gel of reconstituted basement membrane and differentiate to secrete milk proteins [1, 2]. However, when the same cells are cultured on tissue culture plastic, they rapidly suspend their milk production. Hence, normal tissue function strongly depends on its contextual microenvironment. Similar to its control over the physiological processes in normal tissue, the microenvironment exerts regulatory control during tumour development and progression [3, 4]. In the state of the art, tumours are considered organ-like systems consisting of proliferating tumour cells and of various types of resident and recruited stromal cells that affect tumour growth and malignant behaviour [5]. On one hand, a variety of cells of the tumour stroma, including fibroblasts, endothelial cells, pericytes and immune cells, have been shown to support tumour cell proliferation, tumour angiogenesis, escape from immune surveillance and metastasis [6–9]. On the other hand, certain tumour microenvironments restrict tumour progression by repressing tumour cell migration and metastatic dissemination [10]. While the contributions of cancer-associated fibroblasts (CAFs) [11] and tumour-infiltrating immune cells [12] have been largely studied, the role of endothelial cells as regulators of cancer cell behaviour is poorly understood.

Endothelial cells are the major players in angiogenesis, which is one of the hallmarks of cancer progression [13, 14]. A critical step in this process is the activation of quiescent endothelial cells by pro-angiogenic growth factors, referred to as “the angiogenic switch”, which induces a cell biological programme leading to the formation of new blood vessels [15–17]. Angiogenesis has been proposed to be rate limiting for tumour growth, and anti-angiogenic therapies have been established and are routinely used in clinics to treat cancer; however with rather limited efficacy [18]. Endothelial cells have also attracted attention as important regulators of organogenesis and as niches for stem cell maintenance in various tissues, such as brain, liver and pancreas [19–22]. In normal tissue, endothelial cells are critical for tissue homeostasis [23]. They are among the longest-living cells in the body remaining quiescent over years. Normal quiescent endothelial cells suppress local hyperplasia, angiogenesis and inflammation, yet they enhance these processes when they are dysfunctional. Endothelial cells might exert a similar effect during tumour growth and progression, not simply by serving as static structural cells of perfused vessels but by actively modulating the tumour microenvironment and thus tumour cells, which may have profound effects on cancer fate. For example, several tumour-promoting factors originating from endothelial cells have been shown to increase cancer cell proliferation [24].

Here, we speculate that endothelial cells depending on their physiological state may also be able to affect tumour cell invasion and migration by releasing stimulatory or inhibitory factors. With the demonstration of the anti-migratory effect of endothelial cell-derived nidogen-1 on SK-BR-3 breast cancer cells we add nidogen-1 to the regulators of endothelial control over cancer cell migration.

## Methods

### Materials and reagents

The human anti-Paxillin antibody was purchased from BD (cat. Num. 610,052), the anti-ZO-1 from Zymed (cat. Num. 617,300), the anti-FN1 and anti-GAPDH from Sigma (cat. Num. F-3648, cat. Num. G-8795), anti-Actin from Santa Cruz (cat. Num sc-1616), anti-VE-Cadherin from ABcam (ab33168), anti-Nidogen-1 from R&D (cat. Num. MAB2570–100), anti-pSTAT3 and anti-Stat3 from Cell Signaling (cat. Num. 9131, 9132), anti-CD31 from BD Pharmigen (cat. Num. 550,274), anti-phospho Histone 3 from Millipore (cat. Num. 06–570). Fluorescently labeled secondary antibodies and anti-Phalloidin-568 were purchased from Invitrogen, DAPI was obtained from Sigma. FLLL31 was purchased from Sigma (cat. Num. F9057). RhNID1 (cat. Num.: 2570-ND-050) and rhBGN1 (cat. Num. 2667-CM-050) were purchased from R&D, Minneapolis, MN, USA.

### Cell culture

Primary human umbilical vein endothelial cells (HUVECs; PromoCell, Heidelberg, Germany) and primary human dermal microvascular endothelial cells (HDMECs; PromoCell, Heidelberg, Germany) were expanded in endothelial growth medium (EGM) and supplements (Lonza, Basel, Switzerland) on 1.5% gelatine-coated plates. The cancer cell lines SK-BR-3, MCF7, PC-3 and H460 were cultured in DMEM with 10% FBS (Sigma Aldrich, Buchs, Switzerland).

To prepare endothelial cell-conditioned medium (EC-CM), confluent HUVEC monolayers were detached, and the cells were plated onto new cell culture dishes of the same diameter in EGM. To prepare conditioned medium from confluent HUVEC 9/10 of the total cell number was seeded for expansion. To obtain conditioned medium from subconfluent HUVEC 1/10 of the total cell number was plated. After 24 h of culturing, medium was changed with conditioning medium (M199 medium (Sigma Aldrich, Buchs, Switzerland) containing 2% FBS supplemented with 1% penicillin, 0.1% glutamine, 4 μg/ml bovine pituitary extract (Thermo Fisher, Waltham, MA, USA) and 8 U/ml heparin). After a conditioning time of 24 h the supernatant was collected, cell debris were removed and the conditioned medium was stored at − 80 °C. For the experiments the cancer cell lines were expanded for 24 h, then the expansion medium was removed and the cancer cells exposed to the HUVEC conditioned medium for 4 days.

### Immunofluorescence microscopy analysis

Cells, plated and treated on glass coverslips, were washed and fixed in 4% PFA for 20 min at RT, permeabilized with 0.5% NP40 in PBS for 5 min, washed and blocked with 3% BSA in PBS-Triton for 1 h at RT. The cells were then incubated with the primary antibody over night at 4 °C. Fluorescently labeled secondary antibodies were added together with DAPI 1 μg/ml for 1 h in the dark. Cells were washed 5 times, mounted and imaged on a fluorescent microscope (Leica DMI4000).

### siRNA interference

Endothelial cells were transfected with 4 siRNAs for each target (siGENOME Human SMARTpool, Dharmacon Lafayette, Colorado, USA) with Lipofectamine 3000 (Invitrogen, Thermo Fisher Waltham, MA, USA) according to the manufacturer’s instructions. The siRNA sequences are as follows: NID1: GGGCGAACCUGCUAUGAUA, GAAGGUUUAUUAUCGAGAA, UAACCUGGAUCGAAUAGAA, and CCUUCAUAACUGCGACAUA; BGN: GGAGAACAGUGGCUUUGAA, UGAAUGAACUCCACCUAGA, CCAAAGAGAUCUCCCCUGA, and GAACAACGACAUCUCCGAG; HSPG2: GCGCUGCGAUGGUGACUUU, CAACACACACCACGAGCUA, GAGCUAUGUGAAUGCAAU, and ACGGUGGGAAGUUGCGAUA; CLU: GAUAAAGACUCUCAUAGAA, GAAAGAGGAUGCCCUAAAU, GGAAGUAAGUACGUCAAUA, and GUAGAAGUCUCCAGGAAGA; C1QTNF5: GCGAAUCCAUUGCCUCUUU, UGAACGAGCAGGGACAUUA, GGGCCAGCCUGCAGUUUGA, and UGACUACAUUGGCAUCUAU; CYR61: GGGCAGACCCUGUGAAUAU, GGCCAGAAAUGUAUUGUUC, GGUCAAAGUUACCGGGCAG, and GCAGCAAGACCAAGAAAUC; TIE1: GGGAAGCCUCCUACCCUUA, GAAGUUCUGUGCAAAUUGG, CAACAUGGCCUCAGAACUG, and UCGAAACUGUGACGAUGAA; VWF: GGACAGAUCAUGACACUGA, GGAAGACCCUGUGGACUUU, GAAGAGGCCUGCACUCAGU, and GGUCACAUCUUCACAUUCA. After 72 h, the transfection medium was changed, and a second transfection was performed. Twenty-four hours after the second siRNA transfection, the cells were used for experiments.

### Migration and transendothelial migration assay

A total of 50′000 SK-BR-3 cells were plated into Boyden-Chamber inserts with 8 μm pore size (BD Falcon, Corning Tewksbury MA, USA) in 200 μl M199 (Sigma Aldrich, Buchs, Switzerland) 2% FBS medium; the bottom chamber was filled with 800 μl of M199 20% FBS medium. After a migration time of 18 h, the cells were fixed with 4% paraformaldehyde in PBS. Cells that had not crossed the membrane were removed, and cells on the bottom of the insert membrane were stained with DAPI (1 μg/ml), visualized with a Leica DMI4000 fluorescence microscope and quantified using ImageJ software. The trans-endothelial migration assay was performed as previously described [25]. Briefly, a total of 15′000 CellTrace CFSE (Invitrogen, Thermo Fisher Waltham, MA, USA)-labelled SK-BR-3 cells were seeded onto a confluent HUVEC monolayer and incubated over 48 h in 5% CO_2_ at 37 °C without a serum gradient. After removal of non-migrated cells, cells that crossed the HUVEC monolayer were fixed with 4% paraformaldehyde, visualized and quantified as described above. Immunofluorescence and immunoblotting were performed as previously described [26]. All experimental conditions were tested and analysed in three replicates and each condition was tested in biological triplicates.

### RNA isolation and RT-PCR

Total RNA was prepared with Tri-Reagent (Sigma Aldrich, Buchs, Switzerland) and reverse-transcribed with ImProm-II™ reverse transcriptase (Promega, Madison, Wisconsin, USA). Transcripts were quantified using SYBR-green PCR Master Mix in a StepOnePlus PCR system (Applied Biosystems, Foster City, California, USA). Real-time PCR reactions were performed in triplicate, and fold-induction was calculated using the comparative Ct method (ΔΔCt) normalized to ribosomal protein L19 expression. The following primers (sequence 5′-3′) were used: human nid1: TCTACGTCACCACAAATGGCA; human hspg2: GTGTGGTGTTCATCAAGGAGC; human nid2: GAAACGCAGTATGTGGACTATGA; human cyr61: GGTCAAAGTTACCGGGCAGT; human vwf: CCGATGCAGCCTTTTCGGA; human clu: CCAATCAGGGAAGTAAGTACGTC; human c1qtnf5: AACGAGCAGGGACATTACGAC; human tie1: AAGCAGACAGACGTGATCTGG; human bgn: CAGTGGCTTTGAACCTGGAG; and human rpl: GATGCCGGAAAAACACCTTG.

### Immunoblotting analysis

Cells were lysed with 0.5 M Tris-Hcl ph 6.8, 10% SDS, glycerol and the lysates boiled for 5 min. Lysates were normalized for equal amount of protein and loaded onto SDS-polyacrylamide gels and transferred to nitrocellulose membranes (Whatman Protran). Blots were sequentially incubated with 5% milk, the primary antibody (1:1000) overnight, and the HRP-labeled secondary-antibody for 1 h at room temperature. Signals were revealed with Upti-Light™ chemiluminescence reagent (Uptima) and detected with X-Ray films.

### Stable isotope labelling by amino acid in cell culture (SILAC) of HUVEC

HUVECs were cultured in custom-made EGM-2 without arginine and lysine (Lonza), with the addition of SILAC amino acids (^13^C_6_ L-arginine and ^2^H_4_ L-Lysine for the “SILAC medium” or ^13^C_6_^15^N_4_ L-arginine and ^13^C_6_^15^N_2_ L-Lysine for the “SILAC heavy”) (Cambridge Isotope Laboratories) until more than 97% of SILAC amino acids were incorporated into proteins.

Labelled “heavy” and “medium” cells were split either sub-confluent or confluent (ratio of sub-confluent to confluent being 1:20) for 3 days (until the confluent cells had reached 100% confluency), washed in PBS with Ca^2+^ and Mg^2+^, and incubated in EBM-2™ medium (Lonza, Basel, Switzerland) for 4 h. The supernatants were collected and spun at 4 °C (300 g for 10 min, followed by 2000 x g for 10 min and 10,000 x g for 30 min). The cells were counted and the “heavy” supernatant coming from the confluent cells was pooled together with the “medium” supernatant coming from the sub-confluent cells (forward experiment) and vice versa (reverse experiment), adapting the volume to the corresponding cell number. Proteins were extracted using Strataclean resin (Agilent Technologies) as previously described [27], dissolved in 4x sample buffer (NuPAGE LDS loading buffer, Life Technologies) supplemented with 0.1 M DTT and separated on a 4–12% NuPAGE Novex Bis-Tris gel (Life Technologies). Each gel lane was cut in seven slices and proteins were in-gel digested with trypsin. Peptides were loaded onto Empore-C_18_ StageTips and eluted with 80% ACN and 0.5% acetic acid.

### Mass spectrometry (MS) analysis of secretome

Digested peptides were separated by nanoliquid chromatography (Easy nLC, Thermo Fisher Scientific) coupled on line to a linear trap quadrupole (LTQ)-Orbitrap Elite mass spectrometer (Thermo Fisher Scientific) via a nanoelectrospray ion source (Nanospray Flex Ion Source, Thermo Fisher Scientific). Peptides were loaded onto a 20 cm fused silica emitter (New Objective) packed with C_18_-AQ, 1.9 μm resin (Dr Maisch GmbH) and eluted with 5–25% solvent (80% ACN, 0.5% acetic acid) over 90 min (200 nl/min). Full scan MS spectra were acquired in the Orbitrap analyzer with a resolution of 120,000 at 400 Th, and a target value of 10^6^ charges. The 10 most intense ions were selected for high collision dissociation fragmentation with a target value of 40,000 charges and acquired in the Orbitrap with resolution of 15,000 at m/z 400 Th. Data were acquired with Xcalibur software (Thermo Fisher Scientific).

### MS data quantification and analysis

The relative quantification based on SILAC labelling was performed by processing the RAW MS files with MaxQuant version 1.4.1.6 [28]. Proteins and peptides were identified using the Andromeda search engine [29] against the human UniProt database (release-2012 01, 88,847 entries). To search for precursor and fragment ions, an initial maximal mass deviation of 7 ppm and 20 ppm, respectively, was required. Trypsin with full enzyme specificity and only peptides with a minimum length of 7 amino acids were selected. A maximum of two missed cleavages was allowed. Carbamidomethylation (Cys) was set as fixed modification, while Oxidation (Met) and N-acetylation as variable modifications. For protein and peptide identification, we required a maximum false discovery rate (FDR) of 1%. The “requantify” option was enabled.

### Data analysis and normalization

Statistical analysis and annotation of the MS data were performed using the Perseus module of MaxQuant version 1.4.17.2. The reverse and contaminant hits from the MaxQuant output files were excluded from the analysis. Only proteins identified with at least 1 unique peptide and quantified with a minimum of two ratio counts were considered for the analysis.

For each experiment (forward and reverse), the SILAC ratios (sub-confluent/confluent cells) were transformed using the binary logarithm (log2) and normalised by subtracting the median value. Proteins were considered up or down-regulated if the SILAC ratio was higher than one standard deviation from the mean of the calculated ratios in both replicate experiments. Protein annotations were added based on the Uniprot ID of each entry (GO and KEGG categories).

### Human phospho-kinase antibody array

The protein lysates from 10′000’000 cells were analysed in a sandwich immunoassay with membrane-bound capture antibodies and biotinylated phospho-specific detection antibodies on the human phospho-kinase antibody array (ARY003B, R&D System, Minneapolis, MN, USA) according to the manufacturer’s instructions. The spot signals were quantified using ImageJ software.

## Results

### Endothelial cells influence cancer cell invasion and migration

**Fig. 1:**
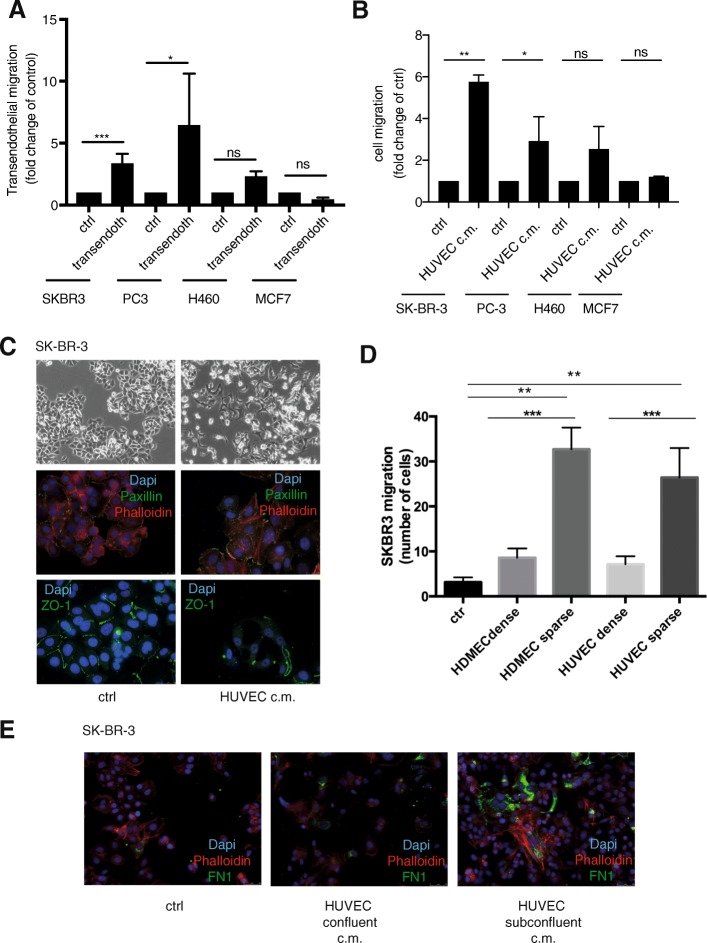
Endothelial cells influence the phenotype and migratory potential of cancer cells. **a** CFSE fluorescently labelled cancer cell lines were plated on a tight HUVEC monolayer on the inner surface of a Boyden migration chamber for migration through the endothelial cell layer. After 48 h, the cells remaining in the insert of the chamber were removed, and the number of migrated cancer cells was quantified by fluorescence microscopy and compared to its own control (migration in absence of endothelial monolayer) . All experimental conditions were tested in three replicates and each condition in biological triplicates. (***p ≤ 0.0001, *p ≤ 0.05 versus control by unpaired student’s t-test, error bar: standard error of the mean). **b** Different cancer cell lines were exposed to human umbilical vein endothelial cell (HUVEC)-conditioned medium for 4 days. After exposure to the HUVEC supernatant, cancer cell migration was evaluated using a Boyden chamber migration assay. The migration rate of each cell line exposed to the HUVEC-conditioned medium was compared to its own control in non-conditioned medium (**p ≤ 0.01, *p ≤ 0.05 versus ctrl by unpaired student’s t-test). **c** SK-BR-3 cell morphology was visualized by phase contrast imaging (top) after exposure to unconditioned or HUVEC-conditioned medium. Expression of actin stress fibres (phalloidin), focal adhesions (paxillin) and tight junctions (ZO-1) was analysed by immunofluorescence staining (middle, bottom). **d** SK -BR-3 cells were exposed to unconditioned (ctrl) or conditioned media derived from confluent or subconfluent HUVEC and HDMEC cultures for 4 days. The effect on the migration of SK-BR-3 cells was evaluated using a Boyden chamber assay (**p ≤ 0.05, ***p ≤ 0.001 versus ctrl by unpaired student’s t-test, error bar: standard error of the mean). **e** Expression of fibronectin (FN1) was detected by immunofluorescent staining of SK-BR-3 cells after 4 days of exposure to unconditioned (ctrl) or conditioned medium derived from confluent or subconfluent HUVEC cultures

We set out to elucidate the influence of endothelial cells on the regulation of cancer cell invasion and migration. We first employed an in vitro co-culture system where cancer cell lines were cultured on a tight layer of commercially available primary human endothelial cells. Upon co-culturing several cell lines started to trans-migrate the endothelial cell layer (Fig. 1a; Additional file 1 Figure S1A). To dissect the mechanisms and to clarify whether secreted factors from endothelial cells are sufficient to influence the migratory potential of the cancer cells without cell-cell contact, we pre-exposed the cancer cell lines to human umbilical vein endothelial cells conditioned medium (HUVEC-CM) for 4 days. Upon exposure to HUVEC-CM, distinct cancer cell lines exhibited a significantly increased ability to migrate following a serum gradient in a modified Boyden-chamber assay (Fig. 1b). The most significant increase in cell migration could be seen with SK-BR-3 human breast cancer cells, and we decided to use this cell line as a model system for further experimentation. PC-3 prostate cancer cells showed the same response to HUVEC-CM (Fig. 1a, b), while MCF7 breast cancer cells and H460 lung cancer cells did not show any change in their migratory potential.

We next assessed whether the observed increased migratory potential of SK-BR-3 cells was associated with changes in cell morphology and expression of differentiation markers. After exposure to HUVEC-CM, SK-BR-3 cells showed a more elongated morphology with an increased number of membrane protrusions (Fig. 1c, upper panel). Additionally, these cells displayed an increase in focal adhesions analysed by paxillin staining and a cytoskeletal re-organization of epithelial cortical actin to mesenchymal stress fibres as shown by phalloidin staining (Fig. 1c, middle panel). Finally, the epithelial cobblestone growth pattern of the SK-BR-3 cells was disrupted, and tight junctions were dissolved as visualized by the loss of ZO-1 from the plasma membrane (Fig. 1c, lower panel). These findings suggest that the factors secreted by cultured endothelial cells induced an epithelial-mesenchymal transition (EMT)-like process in SK-BR-3 cells that is associated with increased SK-BR-3 cell migration and invasion, hallmarks of cancer cell aggressiveness [24]. While in control medium MCF-7 shows an epithelial morphology similar to SK-BR-3, the change of morphology and the ability to migrate could not be observed in MCF-7 upon exposure to HUVEC-CM (data not shown).

The tumour stromal microenvironment plays a dual role either as a promoter [30] or as an inhibitor of cancer progression [31]. Endothelial cells might have a central regulatory function in tumour-stromal interaction depending on their physiological state. Tumour-associated endothelial cells [32, 33] are distinct from normal endothelial cells in healthy tissue [34]. While normal endothelium stays in a quiescent state, the proliferation rate of tumour-associated endothelial cells is dramatically increased [32]. We mimicked these distinctions by defining the culture density of HUVECs in vitro [35]. The quiescent endothelium of normal blood vessels is best represented by a confluent layer of cultured endothelial cells. The activated, proliferating and sprouting endothelium typical of active angiogenesis is best embodied by a proliferating, subconfluent endothelial cell culture [36]. We hypothesized that the quiescent and the proliferating endothelial cell states could differentially affect the migratory potential of cancer cells. To test this hypothesis, supernatants were prepared from human dermal microvascular endothelial cells (HDMEC) and from HUVECs under confluent or subconfluent conditions (Additional file 1 Figure S1B) and added to SK-BR-3 cells for 4 days. Compared to SK-BR-3 cells cultured in unconditioned control medium, the supernatants from HDMECs and HUVECs increased the migratory potential of SK-BR-3. Notably, SK-BR-3 cells treated with supernatants of subconfluent HDMECs and HUVEC cultures had a significantly greater migratory potential than cells exposed to supernatants of confluent cultures (Fig. 1d). Consistent with the induction of a spindle shape cellular phenotype, deposition of fibronectin, a component of the extracellular matrix important for attachment and migration, by SK-BR-3 cells was higher after exposure to supernatants of subconfluent HUVECs than of confluent HUVECs (Fig. 1e).

These findings show that endothelial cells secrete factors that induce a change of morphology of distinct cancer cells such as SK-BR-3 in parallel with the induction of an increased migratory potential. Moreover, these factors are dependent on the growth and activation status of endothelial cells. Since HDMEC and HUVEC supernatant had a very similar effect on the migratory ability of SK-BR-3 cells we decided to focus further experiments on the interaction of SK-BR-3 with HUVEC.

### Endothelial-derived nidogen-1 inhibits migration of SK-BR-3 cells

**Fig. 2:**
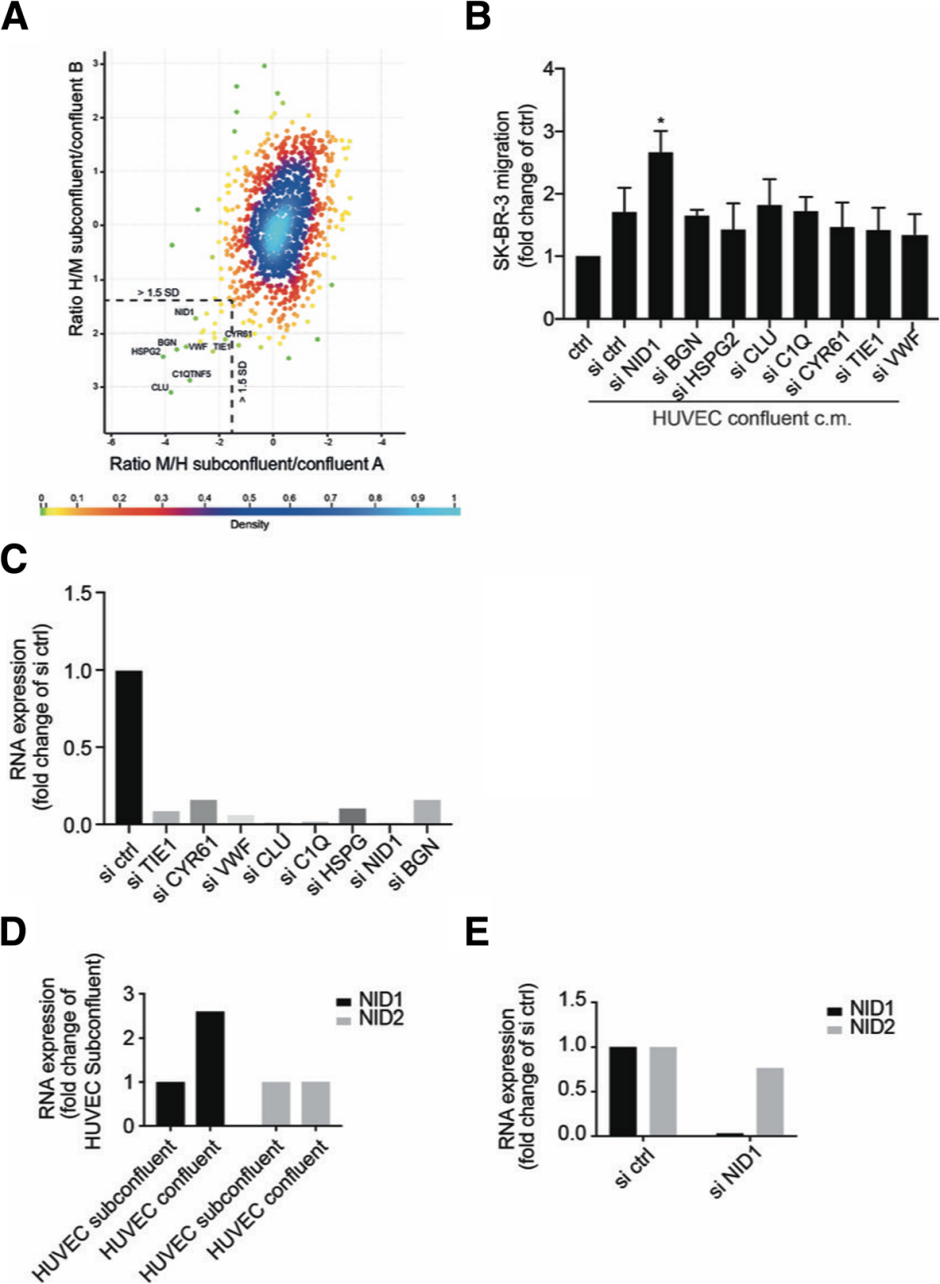
Identification of candidate proteins by SILAC MS proteomic analysis of the endothelial secretome. **a** The protein levels in confluent and subconfluent HUVEC cells were accurately measured with SILAC using the MaxQuant software. The proteins are depicted in a scatter plot. Highlighted in the square are the proteins with a SILAC ratio ≥ 1.5 SD from the mean of the calculated ratio in each replicate experiment, A and B. These proteins were secreted at higher levels by confluent HUVEC. **b** The candidate proteins identified by SILAC MS analysis were silenced with siRNAs in HUVECs kept under confluent growth conditions. The migratory potential of SK-BR-3 cells exposed to conditioned medium of HUVECs with silenced proteins was evaluated using a Boyden chamber migration assay and compared to controls (*p ≤ 0.05 versus ctrl by unpaired student’s t-test). **c** The efficiency of siRNA knockdown was analysed for each candidate gene that was identified by SILAC. The mRNA expression levels were evaluated by quantitative RT-PCR analysis of confluent HUVECs after siRNA-mediated knockdown of the respective gene. **d** Gene expression of NID1 and NID2 was evaluated by quantitative RT-PCR analysis of HUVECs plated in a confluent or subconfluent monolayer. **e** Gene expression of NID1 and NID2 was determined by quantitative RT-PCR analysis of HUVECs plated in a confluent monolayer after siRNA-mediated knock-down of NID1

To identify factors released from endothelial cells that control cancer cell migration, we compared the secretome of confluent with that of subconfluent HUVEC cultures by MS-SILAC proteomics [27, 37] (Fig. 2a, Additional file 2 Table S1). The levels of the vast majority of the proteins in the two conditioned media were similar. Only eight proteins were found at significantly higher levels in the supernatants of confluent compared to subconfluent HUVEC cultures. These proteins were nidogen-1, biglycan, hspg2, clusterin, complement c1q tumour necrosis factor protein 5, Cyr61, Tie1 and von Willebrand factor. Since conditioned medium from confluent HUVECs induced less migration of cancer cells than medium from subconfluent HUVECs, we suspected the identified proteins to be potential repressors of cancer cell migration. We tested this hypothesis by siRNA-mediated depletion of each candidate gene in HUVECs. Supernatants of the target gene-depleted HUVECs were applied to SK-BR-3 cells for 4 days before testing the SK-BR-3 cell migratory potential. Among the eight candidates only the downregulation of nidogen-1 expression in confluent HUVECs was able to significantly increase cancer cell migration (Fig. 2b). Depletion of the target gene was shown by real-time-PCR (Fig. 2c). These results suggest that nidogen-1 might be responsible for the anti-migratory activity in the supernatant of confluent HUVECs.

Nidogen-1 is a member of the nidogen family of basal membrane glycoproteins. Both nidogen-1 and nidogen-2 are essential components of the basal membrane that structure it by connecting networks formed by collagens and laminins. Furthermore, nidogen-1 and nidogen-2 play a role in the interactions of the extracellular matrix with the cells [38].

**Fig. 3:**
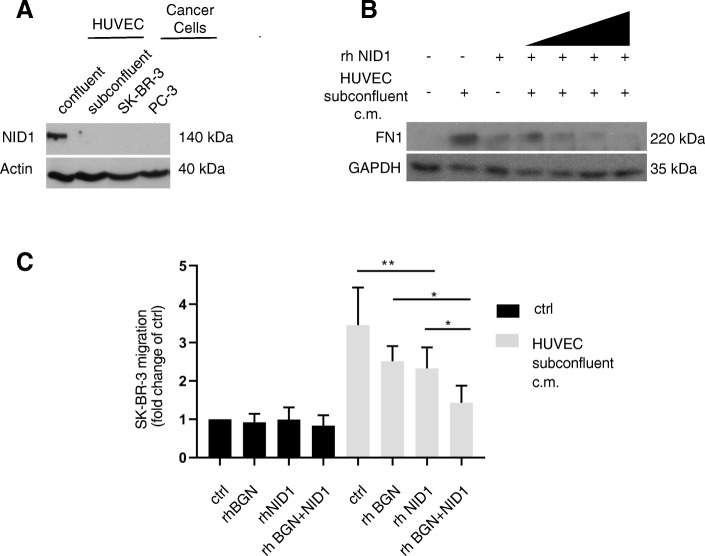
Nidogen-1 blocks endothelial-induced migration of cancer cells. **a** The endogenous expression of nidogen-1 (NID1) protein was determined by western blot analysis in HUVECs kept under confluent or subconfluent growth conditions and in the cancer cell lines SK-BR-3 and PC3. Actin was used as a loading control. **b** Fibronectin-1 (FN1) expression was evaluated by immunoblotting analysis in SK-BR-3 cells exposed to conditioned medium derived from subconfluent HUVECs for 4 days or with increasing concentrations of recombinant nidogen-1 (0, 0.01, 0.05, 0.2, and 0.5 μg/ml). GAPDH was used as a loading control. **c** SK-BR-3 cells were treated for 4 days with unconditioned medium or conditioned medium derived from subconfluent HUVECs with or without recombinant human nidogen-1 (rhNID1 1 μg/ml), with or without human biglycan (rhBGN 1 μg/ml) or in combination. After treatment, the migratory capacity of the cells was determined using a modified Boyden chamber migration assay (**p ≤ 0.01, *p ≤ 0.05 versus control by unpaired student’s t-test)

In our experimental system, only the expression of nidogen-1 was dependent on the HUVEC growth and activation status and depletion of nidogen-1 was not compensated by over-expression of nidogen-2 (Fig. 2d, e). To assess whether nidogen-1 was exclusively expressed by endothelial cells, we tested its protein expression in confluent and subconfluent HUVECs and in SK-BR-3 and PC3 cancer cells (Fig. 3a). Confirming the MS-SILAC analysis, confluent HUVECs expressed significantly higher amounts of nidogen-1 than subconfluent HUVECs. Moreover, the tested cancer cells did not express nidogen-1 at levels comparable to HUVECs. These data raise the possibility that the inhibitory effect of confluent HUVECs on SK-BR-3 migration could depend on the presence of nidogen-1.

The secretion of fibronectin, a known marker of EMT, was increased in SK-BR-3 cells cultured with conditioned medium of subconfluent HUVEC (Fig. 1e, Fig. 3b). However, addition of recombinant nidogen-1 reversed the expression of fibronectin in a dose-dependent manner (Fig. 3b). Furthermore, the pro-migratory effect of the conditioned medium of subconfluent HUVEC on SK-BR-3 cells was significantly reduced in the presence of recombinant nidogen-1, recapitulating the difference observed between the supernatants of confluent and subconfluent endothelial cells (Fig. 3c).

The basal membrane with nidogen-1 as one of its components serves as a strong barrier for cancer cells. Other basal membrane components, such as hspg2, have already been shown to exert an anti-migratory function [39]. In fact, MS-SILAC analysis revealed hspg2 and biglycan to be more abundant in the supernatant of confluent HUVECs than in that of subconfluent HUVECs (Fig. 2a), but only the depletion of nidogen-1 significantly increased SK-BR-3 cell migration. However, recombinant biglycan reduced cancer cell migration to a similar extent than nidogen-1. Furthermore, the combination of recombinant nidogen-1 and biglycan had an additive effect on SK-BR-3 cell migration (Fig. 3c). These results suggest that nidogen-1 from HUVECs cultured at confluence inhibits SK-BR-3 migration, suggesting that nidogen-1 might play a role in the signalling between endothelial and cancer cells to control cancer cell migration.

### STAT3 signalling is a main driver of endothelial-dependent cancer cell migration

**Fig. 4:**
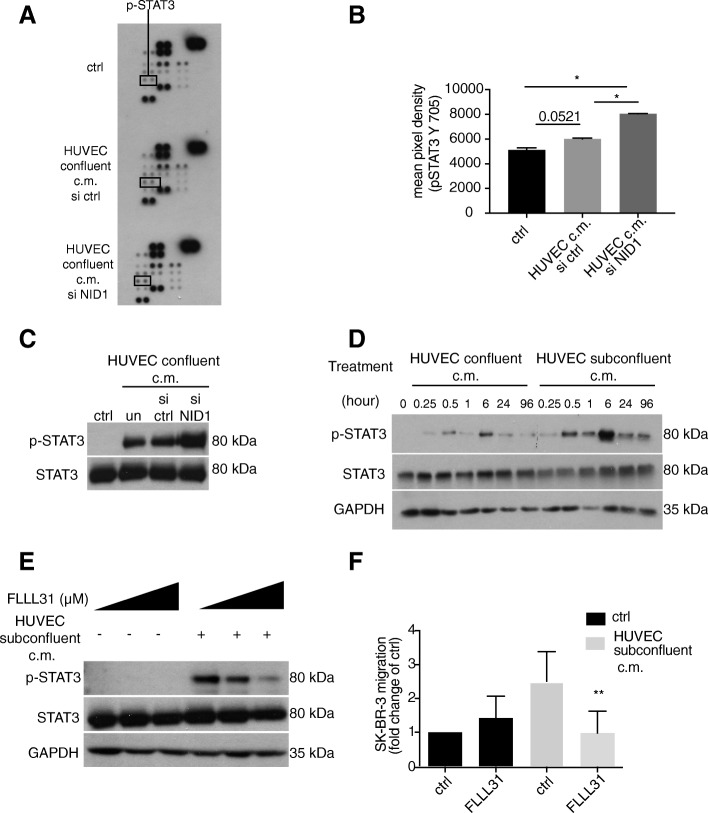
The STAT3 signal promotes endothelial-dependent cell migration and is blocked by NID1. **a** Phospho-protein array was performed on cell lysates of SK-BR-3 cells incubated for 4 days in unconditioned medium (ctrl), or conditioned medium derived from confluent HUVECs transfected with siRNA targeting NID1 (siNID1) or control siRNA. **b** Cell lysates from SK-BR-3 cells, SK-BR-3 cells with control siRNA and SK-BR-3 cells with siRNA knockdown of nidogen-1 were analysed on a phosphoprotein kinase array. The pixel densities of the single dots were quantified by ImageJ software. After subtraction of the background signal, the average of the duplicated spots was plotted (*p ≤ 0.05 versus ctrl by unpaired student’s t-test). **c** The phosphorylation state of STAT3 (Y705) was evaluated in SK-BR-3 cells after 4 days exposure to unconditioned medium (ctrl) or conditioned medium derived from confluent HUVECs treated without (un), with control siRNA (si ctrl) or with NID1 specific siRNA (siNid1). Total STAT3 expression served as loading control. **d** Phosphorylation and total levels of STAT3 in SK-BR-3 cells were evaluated by immunoblotting analysis after incubation with conditioned media derived from confluent or subconfluent HUVECs for the time points indicated. GAPDH was used as loading control. **e** Immunoblotting analysis of phosphorylated (Y705) and total STAT3 levels in SK-BR-3 cells pretreated with increasing concentrations (0 μM, 0.5 μM, and 1 μM) of the STAT3 inhibitor FLLL31 for 18 h and incubation in conditioned medium derived from subconfluent HUVECs. GAPDH served as loading control. **f** The migratory ability of SK-BR-3 cells was determined using a modified Boyden chamber assay after exposure to conditioned medium derived from subconfluent HUVECs with or without the STAT3 inhibitor FLLL31 (0.5 μM) for 4 days (**p ≤ 0.01 versus ctrl by unpaired student’s t-test)

Cell migration and metastasis is a multistep process that involves the activation of many transcription factors and signalling pathways, mostly triggered by the tumour microenvironment [14, 40]^,^. To determine which signalling pathways could be involved in nidogen-1-dependent migration, we screened a series of known signalling effector proteins for their activation by phosphorylation on a phosphoprotein array. SK-BR-3 cells were exposed to conditioned medium of confluent nidogen-1-depleted HUVECs (siNID1) or to conditioned medium of confluent HUVECs transfected with control siRNA (siCtrl) or to normal growth medium. Cell lysates of SK-BR-3 cells exposed to these three conditions were analysed on a human phosphoprotein array to screen for site-specific phosphorylation of 43 kinases and their effector proteins. Most of the proteins exhibited absent or unchanged phosphorylation levels. However, with the nidogen-1-depleted conditioned medium of confluent HUVECs the pSTAT3 (Y705) signal was markedly increased in SK-BR-3 cells as compared to control supernatants (Fig. 4a-c), suggesting an inhibitory effect of nidogen-1 on STAT3 phosphorylation.

STAT3 signalling has been shown to promote cancer cell migration in response to environmental stimuli [41]. Consistent with the results of the phosphoprotein array, immunoblotting analysis showed that STAT3 phosphorylation levels are higher after the stimulation of SK-BR-3 cells with the supernatant of the subconfluent HUVEC culture than that of the confluent HUVEC culture. A time course displayed increasing phosphorylation over time, with a peak at 6 h (Fig. 4d). These results suggest STAT3 signalling as one of the main inducers of cancer cell migration stimulated by endothelial cells. In fact, blocking STAT3 signalling with the specific JAK/STAT inhibitor FLLL31 inhibited the pro-migratory effect on SK-BR-3 cells induced by stimulation with conditioned medium of subconfluent HUVECs (Fig. 4e, f). This inhibitory effect of FLLL31 with a decrease in SK-BR-3 migration by 67% was very similar to the decrease observed with HUVEC confluent conditioned medium by 70%. The decrease in SK-BR-3 migration by rNID1 compared to migration induced by HUVEC subconfluent conditioned medium was 35.5%. These results show that conditioned medium derived from endothelial cells activates the promigratory STAT3 signalling pathway and stimulates SK-BR-3 migration. Depletion of nidogen-1 from conditioned medium of confluent endothelial cells further enhances STAT3 activation and SK-BR-3 migration reflecting the situation of conditioned medium from subconfluent endothelial cells where nidogen-1 is inherently not expressed. Together the data show that depending on their physiological state endothelial cells exert a regulatory control over the migratory potential of distinct cancer cells and a systematic molecular screen revealed nidogen-1 as a relevant endothelial cell derived inhibitor of migration of these cancer cells.

## Discussion

The microenvironment plays a central role in cancer control [8, 30]. Here, we have delineated in well controllable two-dimensional in vitro experiments how endothelial cells, a main component of the tumour environment, influence important morphological features and functional characteristics of cancer cells, such as EMT-like changes, cell invasion and migration. We show that paracrine signals originating from endothelial cells can lead to increased cell migration and trans-endothelial invasion of distinct cancer cell lines. We have shown the effects of conditioned medium derived from two commercially available primary human endothelial cells, HUVEC and HDMEC, on specific tumor cell lines suggesting a general characteristic of endothelial cells, but we are aware that we did not test the effect of primary tumor derived endothelial cells on cancer cells in an in vivo three-dimensional environment. While several of the tested cancer cell lines responded to the endothelial stimuli with increased migratory potential, others remained unresponsive. This finding suggests that the endothelial microenvironment is not a general modulator of tumour progression but needs to match distinct features of the cancer cells, most likely their transformation status and their responsiveness to EMT-like changes.

Furthermore, we show that the ability of endothelial cells to stimulate or to inhibit cancer cell migration is dependent on the physiological status of endothelial cells. Comparing the secretome of confluent, quiescent HUVECs with that of subconfluent, proliferative HUVECs by MS-SILAC analysis has revealed a number of significantly more abundant proteins in the supernatant of confluent HUVECs, thus representing potential inhibitors of tumour cell migration. Remarkably, basal membrane and extracellular matrix proteins (nidogen-1, biglycan, cyr61, hspg2) were over-represented. Supporting our hypothesis, hspg2 has been previously described as an endothelial cell-derived inhibitor of cancer cell migration and metastasis [39]. However, the actual role of extracellular matrix proteins in cancer cell invasion and migration has remained elusive. For instance, biglycan has been reported to either induce cancer cell invasion [42] or to inhibit metastasis [43, 44]. With this report we show that endothelial cell derived nidogen-1 represses cancer cell migration.

Nidogen-1 belongs to a family of glycoproteins present in the basal membrane of different organs, mainly in blood vessels [45, 46]. The functional effects of nidogen-1 in carcinogenesis however remain conflicting. On one hand, a recent publication describes nidogen-1 as a promoter of metastasis to the lung in a breast cancer and melanoma model and high expression of nidogen-1 to correlate with an unfavourable prognosis in estrogen receptor negative breast cancer [47]. Also in ovarian cancer, nidogen-1 has been shown to promote EMT and metastasis [48]. On the other hand, the *NID1* locus has been described in a genome-wide association study to be linked with the risk of developing melanoma with a decreased expression of nidogen-1 in nevi and melanoma patients [49]. Loss of nidogen-1 by aberrant promoter methylation has also been linked to development of colon and stomach cancer [50], and also in prostate cancer loss of nidogen-1 increased tumour growth and metastasis [51]. In line with these reports showing an inhibitory effect of nidogen-1 on cancer cell migration and metastasis, using gain and loss of function experiments we demonstrate that endothelial derived nidogen-1 is an inhibitor of migration for certain cancer cell types, such as SKBR-3 human breast cancer cells. Since an adequate control protein is difficult to find, we compared the inhibiton of migration by nidogen-1 against HUVEC subconfluent conditioned medium as a control which might be viewed as a limitation of this observation. In parallel with the inhibition of migration the expression of fibronection, a marker for EMT, is decreased in SK-BR-3 upon stimulation with nidogen-1. While stromal derived nidogen-2 has previously been shown to repress the number of metastases in a melanoma model [52] and its expression has also been shown to inhibit metastasis in nasopharyngeal and oesophageal carcinoma [53], equal expression of nidogen-2 in confluent and subconfluent HUVEC cells indicates that nidogen-2 does not play any role in the endothelial control of SK-BR-3 breast cancer cell migration. This suggests that the influence of the two nidogen isoforms might be specific for the cancer cell type and should be analysed separately with regard to the respective tumour-stromal context.

We further show that conditioned medium derived from endothelial cells activates the promigratory STAT3 signalling pathway and stimulates SK-BR-3 migration. These effects are further enhanced in the absence of nidogen-1, either by inherent absence of nidogen-1 in conditionend medium from subconfluent endothelial cells or by siRNA-mediated depletion of nidogen-1 from endothelial cells. STAT3 signalling is well known to be activated in cancer [54, 55] and is specifically involved in EMT, in the acquisition of a stem-cell-like phenotype and in defining the premetastatic niche [56]. In our experimental system, STAT3 is the main signal transducer leading to endothelial induced tumour cell migration, as inhibition with the STAT3 signalling inhibitor FLLL31 is sufficient to repress endothelial cell-dependent migration of SK-BR-3 cells. However, how STAT3 signalling and cancer cell migration are induced by subconfluent HUVEC medium, how nidogen-1 represses STAT3 phosphorylation and thus its signalling effector role, and whether such repression is the only mode of action of nidogen-1 in repressing cancer cell migration remain to be resolved. Conditioned medium derived from subconfluent or confluent HUVECs contains a variety of growth factors, including several members of the EGF and FGF families (data not shown). Nidogen-1 thus might interfere with the ligand-receptor binding of pro-migratory factors secreted by endothelial cells or by direct signalling to the recipient cells. These mechanisms warrant further investigation.

## Conclusion

With the identification of nidogen-1 as an inhibitor of cancer cell migration, we characterized an endothelial cell-derived regulator critical for malignant tumour progression. Proliferative endothelial cells are able to release factors to induce cancer cell migration with the activation of pro-migratory signals, such as STAT3. Quiescent endothelial cells exert control over cancer cell migration by releasing inhibitory factors such as nidogen-1. It appears that these interactions are specific for distinct tumour types. Published evidence [47] and our data suggest that nidogen-1 might play a dual role in cancer cell migration depending on the tumour type and the microenvironmental context. The molecular details of the crosstalk between endothelial cells and cancer cells, besides angiogenesis, offer the opportunity to discover new therapeutic targets and to develop new strategies for innovative therapies.

## Additional files

Additional file 1: **Figure S1.** Trans-endothelial migration assay. (A) Schematic representation of the trans-endothelial migration assay. (B) Expression of VE-cadherin was evaluated by immunofluorescence microscopy analysis of HUVECs plated in a confluent or subconfluent monolayer. (PDF 10275 kb)

Additional file 2: **Table S1.** List of proteins identified by MS-SILAC. Proteins identified with at least 1 unique peptide and quantified with a minimum of two ratio counts were considered for the analysis. For each experiment (forward and reverse), the SILAC ratios (sub-confluent/confluent cells) are given. NaN = Not a Number. (XLSX 328 kb)

## Abbreviations

ACN: Acetonitril
Ca^2^: Calcium
CAF: Carcinoma associated fibroblast
CFSE: Carboxyfluoresceinsuccinimidylester
CO^2^: Carbon dioxide
Ctrl: Control
DAPI: 4′,6-Diamidin-2-phenylindol
DMEM: Dulbecco Modified Eagle Medium
DTT: Dithiothreitol
EC-CM: Endothelial-cell conditioned medium
EGF: Epidermal growth factor
EGM: Endothelial cell growth medium
EMT: Epithelial-mesenchymal transition
FBS: Fetal bovine serum
FDR: False discovery rate
FGF: Fibroblast growth factor
FLL31: Potent inhibitor of STAT3
g: Gravitational constant
h: Hour
HDMEC: Human dermal microvascular endothelial cell
HUVEC: Human umbilical vein endothelial cell
HUVEC-CM: Human umbilical vein endothelial cell-conditioned medium
Mg^2^: Magnesium
min: Minute
mRNA: Messenger ribonucleic acid
MS: Mass spectrometry
NaN: Rot a number
NID1: Nidogen1 gene
PBS: Phosphate buffered saline
PCR: Polymerase chain reaction
ppm: Parts per million
RT-PCR: Reversetranscriptase-polymerase chain reaction
SDS: Sodium dodecyl sulfate
SILAC: Stable isotope labeling by amino acids in cell culture
siRNA: Small interfering ribonucleic acid
Th: Thomson
μm: Micrometer
μM: Micromolar

## References

[1] Barcellos-Hoff MH, Aggeler J, Ram TG, Bissell MJ (1989). Functional differentiation and alveolar morphogenesis of primary mammary cultures on reconstituted basement membrane. Development..

[2] Lin CQ, Bissell MJ (1993). Multi-faceted regulation of cell differentiation by extracellular matrix. FASEB J.

[3] Bissell MJ, Radisky D (2001). Putting tumours in context. Nat Rev Cancer.

[4] Weinberg RA. Coevolution in the tumor microenvironment. Nat Genet. 2008;40(5). 10.1038/ng0508-494 18443582

[5] Hanahan D, Coussens LM (2012). Accessories to the crime: functions of cells recruited to the tumor microenvironment. Cancer Cell.

[6] Egeblad M, Nakasone ES, Werb Z (2010). Tumors as organs: complex tissues that interface with the entire organism. Dev Cell.

[7] Qian B-Z, Pollard JW (2010). Macrophage diversity enhances tumor progression and metastasis. Cell..

[8] Joyce JA, Pollard JW (2009). Microenvironmental regulation of metastasis. Nat Rev Cancer.

[9] Kalluri R, Zeisberg M (2006). Fibroblasts in cancer. Nat Rev Cancer.

[10] Nguyen-Ngoc K-V, Cheung KJ, Brenot A, Shamir ER, Gray RS, Hines WC (2012). ECM microenvironment regulates collective migration and local dissemination in normal and malignant mammary epithelium. Proc Natl Acad Sci.

[11] Bhowmick NA, Neilson EG, Moses HL (2004). Stromal fibroblasts in cancer initiation and progression. Nature..

[12] Condeelis J, Pollard JW (2006). Macrophages: obligate partners for tumor cell migration, invasion, and metastasis. Cell..

[13] Folkman J (1971). Tumor angiogenesis: therapeutic implications. N Engl J Med.

[14] Hanahan D, Weinberg RA (2000). The hallmarks of cancer. Cell..

[15] Hanahan D, Folkman J (1996). Patterns and emerging mechanisms of the angiogenic switch during tumorigenesis. Cell..

[16] Hanahan D, Christofori G, Naik P, Arbeit J (1996). Transgenic mouse models of tumour angiogenesis: the angiogenic switch, its molecular controls, and prospects for preclinical therapeutic models. Eur J Cancer.

[17] Baeriswyl V, Christofori G (2009). The angiogenic switch in carcinogenesis. Semin Cancer Biol.

[18] Aalders KC, Tryfonidis K, Eb S, Cardoso F. Anti-angiogenic treatment in breast cancer: facts, successes, failures and future perspectives. Cancer Treat Rev. 2017. 10.1016/j.ctrv.2016.12.009 28088074

[19] Shen Q, Goderie SK, Jin L, Karanth N, Sun Y, Abramova N (2004). Endothelial cells stimulate self-renewal and expand neurogenesis of neural stem cells. Science..

[20] Yin T, Li L (2006). The stem cell niches in bone. J Clin Investig.

[21] Matsumoto K, Yoshitomi H, Rossant J, Zaret KS (2001). Liver organogenesis promoted by endothelial cells prior to vascular function. Science..

[22] Lammert E, Cleaver O, Melton D (2001). Induction of pancreatic differentiation by signals from blood vessels. Science..

[23] Aird WC (2008). Endothelium in health and disease. Pharmacol Rep.

[24] Sigurdsson V, Hilmarsdottir B, Sigmundsdottir H, Fridriksdottir AJ, Ringnér M, Villadsen R (2011). Endothelial induced EMT in breast epithelial cells with stem cell properties. PLoS One.

[25] Giannotta M, Benedetti S, Tedesco FS, Corada M, Trani M, D'antuono R, et al. Targeting endothelial junctional adhesion molecule-a/EPAC/Rap-1 axis as a novel strategy to increase stem cell engraftment in dystrophic muscles. EMBO molecular medicine. 2013:e201302520. 10.1002/emmm.201302520 PMC3927958 24378569

[26] Waldmeier L, Meyer-Schaller N, Diepenbruck M, Christofori G (2012). Py2T murine breast cancer cells, a versatile model of TGFβ-induced EMT in vitro and in vivo. PLoS One.

[27] Hernandez-Fernaud JR, Ruengeler E, Casazza A, Neilson LJ, Pulleine E, Santi A (2017). Secreted CLIC3 drives cancer progression through its glutathione-dependent oxidoreductase activity. Nat Commun.

[28] Jr C, Mann M (2008). MaxQuant enables high peptide identification rates, individualized ppb-range mass accuracies and proteome-wide protein quantification. Nat Biotechnol.

[29] Cox J, Neuhauser N, Michalski A, Scheltema RA, Olsen JV, Mann M (2011). Andromeda: a peptide search engine integrated into the MaxQuant environment. J Proteome Res.

[30] Bissell MJ, Hines WC (2011). Why don't we get more cancer? A proposed role of the microenvironment in restraining cancer progression. Nat Med.

[31] Rhim AD, Oberstein PE, Thomas DH, Mirek ET, Palermo CF, Sastra SA (2014). Stromal elements act to restrain, rather than support, pancreatic ductal adenocarcinoma. Cancer Cell.

[32] Dudley AC (2012). Tumor endothelial cells. Cold Spring Harbor perspectives in medicine.

[33] Hida K, Maishi N, Torii C, Hida Y (2016). Tumor angiogenesis--characteristics of tumor endothelial cells. Int J Clin Oncol.

[34] Charalambous C, Hofman FM, Chen TC (2005). Functional and phenotypic differences between glioblastoma multiforme-derived and normal human brain endothelial cells. J Neurosurg.

[35] Dejana E (2004). Endothelial cell-cell junctions: happy together. Nat Rev Mol Cell Biol.

[36] Ghajar CM, Peinado H, Mori H (2013). The perivascular niche regulates breast tumour dormancy. Nat Cell Biol.

[37] Ong S-E, Blagoev B, Kratchmarova I, Kristensen DB, Steen H, Pandey A (2002). Stable isotope labeling by amino acids in cell culture, SILAC, as a simple and accurate approach to expression proteomics. Mol Cell Proteomics.

[38] Pujuguet P, Simian M, Liaw J, Timpl R, Werb Z, Bissell MJ. Nidogen-1 regulates laminin-1-dependent mammary-specific gene expression. J Cell Sci 2000;113 ( Pt 5)(5):849–858. 10.1242/jcs.113.5.849 PMC2933215 10671374

[39] Franses JW, Baker AB, Chitalia VC, Edelman ER (2011). Stromal endothelial cells directly influence cancer progression. Sci Transl Med.

[40] Hanahan D, Weinberg RA (2011). Hallmarks of cancer: the next generation. Cell..

[41] Wu X, Tao P, Zhou Q, Li J, Yu Z, Wang X (2017). IL-6 secreted by cancer-associated fibroblasts promotes epithelial-mesenchymal transition and metastasis of gastric cancer via JAK2/STAT3 signaling pathway. Oncotarget..

[42] Andrlova H, Mastroianni J, Madl J, Kern J, Melchinger W, Dierbach H, et al. Biglycan expression in the melanoma microenvironment promotes invasiveness via increased tissue stiffness inducing integrin-beta1 expression. Oncotarget. 2017. 10.18632/oncotarget.17160 PMC5522114 28476030

[43] Bischof AG, Yüksel D, Mammoto T, Mammoto A, Krause S, Ingber DE (2013). Breast cancer normalization induced by embryonic mesenchyme is mediated by extracellular matrix biglycan. Integr Biol.

[44] Niedworok C, Rock K, Kretschmer I, Freudenberger T, Nagy N, Szarvas T, et al. Inhibitory role of the small leucine-rich proteoglycan biglycan in bladder cancer. PLoS One 2013;8(11):e80084. doi: 10.1371/journal.pone.0080084. PubMed PMID: 24223213; PubMed Central PMCID: PMC3819308. 10.1371/journal.pone.0080084 PMC3819308 24223213

[45] Kohfeldt E, Sasaki T, Göhring W, Timpl R (1998). Nidogen-2: a new basement membrane protein with diverse binding properties. J Mol Biol.

[46] Yurchenco PD, Schittny JC (1990). Molecular architecture of basement membranes. FASEB J.

[47] Aleckovic M, Wei Y, LeRoy G, Sidoli S, Liu DD, Garcia BA, et al. Identification of Nidogen 1 as a lung metastasis protein through secretome analysis. Genes Dev 2017;31(14):1439–1455. doi: 10.1101/gad.301937.117. PubMed PMID: 28827399; PubMed Central PMCID: PMC5588926. 10.1101/gad.301937.117 PMC5588926 28827399

[48] Zhou Y, Zhu Y, Fan X, Zhang C, Wang Y, Zhang L, et al. NID1, a new regulator of EMT required for metastasis and chemoresistance of ovarian cancer cells. Oncotarget. 2017;8(20):33110–33121. doi: 10.18632/oncotarget.16145. PubMed PMID: 28416770; PubMed Central PMCID: PMC5464854. 10.18632/oncotarget.16145 PMC5464854 28416770

[49] Nan H, Xu M, Zhang J, Zhang M, Kraft P, Qureshi AA (2011). Genome-wide association study identifies nidogen 1 (NID1) as a susceptibility locus to cutaneous nevi and melanoma risk. Hum Mol Genet.

[50] Ulazzi L, Sabbioni S, Miotto E, Veronese A, Angusti A, Gafa R, et al. Nidogen 1 and 2 gene promoters are aberrantly methylated in human gastrointestinal cancer. Mol Cancer 2007;6:17. doi: 10.1186/1476-4598-6-17. PubMed PMID: 17328794; PubMed Central PMCID: PMC1831485. 10.1186/1476-4598-6-17 PMC1831485 17328794

[51] Ko CJ, Huang CC, Lin HY, Juan CP, Lan SW, Shyu HY, et al. Androgen-induced TMPRSS2 activates Matriptase and promotes extracellular matrix degradation, prostate Cancer cell invasion, tumor growth, and metastasis. Cancer Res 2015;75(14):2949–2960. doi: 10.1158/0008-5472.CAN-14-3297. PubMed PMID: 26018085. 10.1158/0008-5472.CAN-14-3297 26018085

[52] Mokkapati S, Bechtel M, Reibetanz M, Miosge N, Nischt R (2012). Absence of the basement membrane component nidogen 2, but not of nidogen 1, results in increased lung metastasis in mice. Journal of Histochemistry & Cytochemistry.

[53] Chai AWY, Cheung AKL, Dai W, Ko JMY, Ip JCY, Chan KW (2016). Metastasis-suppressing NID2, an epigenetically-silenced gene, in the pathogenesis of nasopharyngeal carcinoma and esophageal squamous cell carcinoma. Oncotarget.

[54] Wang N, Wei L, Huang Y, Wu Y, Su M, Pang X (2017). miR520c blocks EMT progression of human breast cancer cells by repressing STAT3. Oncol Rep.

[55] Xu Y, Lu S (2014). A meta-analysis of STAT3 and phospho-STAT3 expression and survival of patients with non-small-cell lung cancer. European Journal of Surgical Oncology (EJSO).

[56] Yu H, Lee H, Herrmann A, Buettner R, Jove R (2014). Revisiting STAT3 signalling in cancer: new and unexpected biological functions. Nat Rev Cancer.

