# Supplementary figures and images for: Endothelial cell-derived nidogen-1 inhibits migration of SK-BR-3 breast cancer cells
Source: BMC Cancer. 2019 Apr 4;19:312. doi: 10.1186/s12885-019-5521-8 (PMC6449935; doi:10.1186/s12885-019-5521-8)

# Figure S1

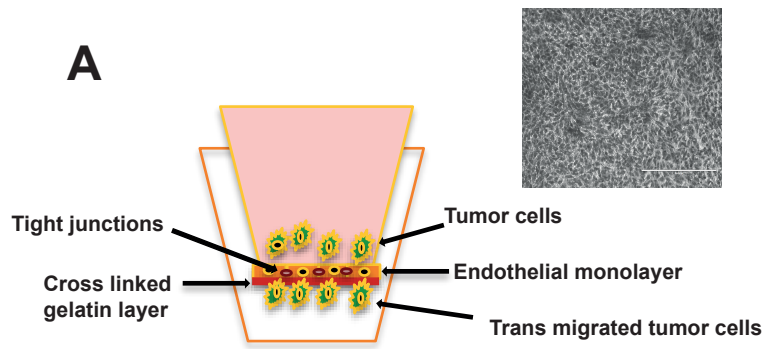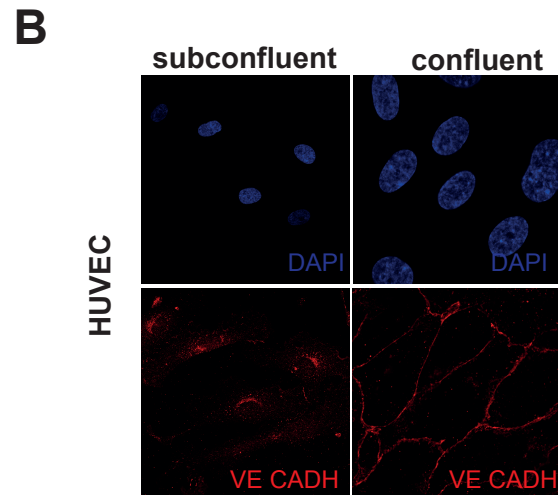

Supplement: Supplementary file 1 — Figure S1. Trans-endothelial migration assay. (A) Schematic representation of the trans-endothelial migration assay. (B) Expression of VE-cadherin was evaluated by immunofluorescence microscopy analysis of HUVECs plated in a confluent or subconfluent monolayer. (PDF 10275 kb) [file 12885_2019_5521_MOESM1_ESM.pdf]
